# Key aroma and smoke components influencing sensory quality of heated tobacco products

**DOI:** 10.3389/fpls.2026.1824692

**Published:** 2026-05-04

**Authors:** Tong Wang, Chao Wang, Jing Yang, Wentao Zhao, Xu Wang, Jianeng Tan, Yuqing Dou

**Affiliations:** 1 Tobacco Research Institute of Chinese Academy of Agricultural Sciences, Qingdao, Shandong, China; 2 Graduate School of Chinese Academy of Agricultural Sciences, Beijing, China; 3 Shanghai New Tobacco Products Research Institute Co. Ltd., Shanghai, China

**Keywords:** aroma component, canonical correlation analysis, heated tobacco products, OPLS-DA, sensory quality, smoke component

## Abstract

Heated tobacco products (HTPs) sensory quality depends jointly on the components of the tobacco raw materials and the smoke. However, the relationships among tobacco leaf aroma components, heated tobacco smoke components and sensory quality remain unclear. In this study, flue-cured tobacco, sun-cured yellow tobacco and sun-cured red tobacco from multiple producing regions were selected as raw materials for HTPs. Aroma components in tobacco leaf powder and key smoke components were determined by chromatographic methods. Orthogonal partial least squares discriminant analysis (OPLS-DA) was used to characterize differences among tobacco types and to screen key components in leaf powder and smoke components. Pearson correlation and canonical correlation analysis (CCA) were applied to explore the relationships between aroma components and sensory quality. Some fatty acids, polyphenols, neutral aroma components and nitrogen-containing components were identified as key components to sensory quality. Palmitic acid and chlorogenic acid were positively correlated with overall sensory scores and negatively correlated with irritancy, dryness, and bitterness. In contrast, citric acid, and several furan and nitrogen-containing components were associated with enhanced negative sensory quality. These key aroma components were associated with most of the variability in HTPs sensory quality, providing a practical basis for raw material screening and product optimization.

## 1 Introduction

Tobacco is a globally significant economic crop, mainly used in the production of traditional tobacco and new tobacco products. The sensory experience during smoking directly determines consumer acceptance and market competitiveness, while the aroma quality of tobacco is the core factors influencing the sensory quality.

HTPs is a new tobacco product that mainly uses external heat source to heat the tobacco materials, the heating temperature is generally below 350°C, and the cigarettes are pyrolyzed rather than burned (Gasparyan et al., 2018), and the production of HTPs and the mechanism of fume and aroma are different from that of traditional cigarettes, so there are large differences in the heated tobacco smoke components between the two (Schaller et al., 2016; Savareear et al., 2019), which have a greater impact on the sensory quality of heated tobacco as well.

Previous studies have examined the relationships between tobacco aroma components and the sensory quality of HTPs. For example, Zhao et al (Zhao et al., 2024) investigated non-flue-cured tobaccos used for HTPs and reported that differential volatile metabolites were positively correlated with smoothness irritancy, dry sensation and cleanliness, and negatively correlated with offensive odor and strength. Liu et al (Liu et al., 2025) proposed an evaluation method for tobacco raw materials used in HTPs based on sensory quality and conventional constituents, and found that total nitrogen and nicotine were strongly correlated with sensory indicators, whereas the correlation between different sugar and sensory indicators was relatively low. Other study (Zhao et al., 2023) has shown that integrated sensory evaluation with non-targeted metabolomics to analyze 5 tobacco varieties for HTPs. and metabolomic analysis revealed that β-damascenone, scopoletin, chlorogenic acids, neochlorogenic acids, flavonol glycosyl derivatives, several lysophospholipids and sugar molecules, were positively associated with sensory quality. In addition, study (Huang et al., 2024) on sun-cured yellow tobacco from different regions of Guangdong Province indicated that sensory quality improved with decreasing sugar and total nitrogen contents and increasing nicotine, organic acids and polyphenols.

From the current research, most research on the sensory quality of tobacco products focused on the influence of origin or chemical components, but there are fewer studies on the influence of aroma components and smoke components on the sensory quality of heated tobacco product.

In this work, flue-cured tobacco, sun-cured yellow tobacco and sun-cured red tobacco from different origins were selected as raw materials. Through various detection methods, obtained the data of tobacco leaves powder aroma components, heated tobacco smoke components and sensory quality scores. Used OPLS-DA, CCA and ANOVA to identify the aroma and heated tobacco smoke components that have a significant affect on the sensory indicators of HTPs and to clarify their contribution directions. The aim is to establish a chemometric framework that links tobacco leaf aroma components, smoke composition and sensory quality, thereby providing guidance for raw material selection and quality improvement of HTPs.

## 2 Materials and methods

### 2.1 Materials

The test materials consisted of middle and upper tobacco leaves collected in 2020–2021 from 10 provinces in China, including Yunnan, Guizhou, Liaoning, Jilin, Hunan, Shandong, Sichuan, Guangxi, Guangdong and Hubei. A total of 42 samples were used: 18 flue-cured tobaccos, 8 sun-cured yellow tobaccos and 16 sun-cured red tobaccos in **Table 1**. The samples were sorted, stemmed, grinded and sieved to produce 200 mesh tobacco powder.

**Table 1:** Basic information of tobacco raw material samples for heated tobacco products preparation in this study.

Type	Number	Origin	Part	Quantities
Flue-cured Tobacco	K1, K2, K3, K4	Yunnan	Upper leaves, Middle leaves	18
	K5, K6, K7, K8	Guizhou		
	K9, K10	Liaoning		
	K11, K12	Jilin		
	K13, K14	Hunan		
	K15, K16	Shandong		
	K17, K18	Sichuan		
Sun-cured Yellow Tobacco	S1, S2, S3, S4	Guangxi	Upper leaves, Middle leaves	8
	S5, S6, S7, S8	Guangdong		
Sun-cured Red Tobacco	S9, S10	Jilin	Upper leaves, Middle leaves, Blended samples of upper and middle leaves	16
	S11, S12, S13	Hubei		
	S14, S15, S16	Liaoning		
	S17	Shandong		
	S18, S23, S24	Yunnan		
	S19, S20, S21	Sichuan		
	S22	Hunan		

### 2.2 Determination of tobacco leaf powder aroma components

The tobacco leaf powder aroma components include non-volatile organic acids, polyphenols, carotenoids and neutral aroma components. The specific detection methods are as follows.

#### 2.2.1 Organic acids

Quantitative detection of organic acids (ug/g): Under acidic conditions, there were esterification and displacement reactions performed to convert all organic acids into methyl ester components. Then, dichloromethane extraction, centrifugal separation, and quantitative analysis by GC/FID were conducted to detect the content of several organic acids (Harvey et al., 1970; Jie et al., 2025).

#### 2.2.2 Polyphenolic components

Quantitative detection of polyphenolic components (mg/g): Referring to the method in reference (Li X. et al., 2021), quantitative detection of polyphenolic components was conducted on the samples, with two parallel detections for each sample.

#### 2.2.3 Carotenoids

Quantitative detection of carotenoids (ug/g): Referring to the method in reference (Xiao et al., 2022), quantitative detection of carotenoids was conducted on the samples, with two parallel detections carried out for each sample.

#### 2.2.4 Neutral aroma components

Semi-quantitative detection of neutral aroma components: Referring to the method in reference (Ding et al., 2013), the neutral flavor components in the samples were detected and analyzed by liquid chromatography-gas chromatography-mass spectrometry (LC-GC-MS) through extraction with a mixed solvent of n-hexane and methyl tert-butyl ether (MTBE).

### 2.3 Determination of heated tobacco smoke components

Instruments and reagents:

Methyl tert-butyl ether (99.8%, Anaqua Corporation, USA); Methyl pentanoate (98%), methyl heptanoate (98.2%), methyl nonanoate (98%), methyl tridecanoate (98.1%), methyl decanoate (98%) (ChemService, USA).

7890B/5977A gas chromatography-mass spectrometry (GC-MS), DB-5MS chromatographic column (30m×0.32mm id×0.25μmdf), DB-WAX chromatographic column (60m×0.32mm id×0.25μmdf) (Agilent, USA), X500E rotary smoking machine (Puffman, China).

Smoke capture method:

Adopted HCL mode, puffing capacity of 55 mL, puffing interval of 30 seconds, puffing duration of 2 seconds, the heating device was FIRAVO H20, and the heating temperature was 350 °C. The determination of particulate matter components in smoke was conducted using the heart-cut two dimensional chromatography-mass spectrometry. Four cigarettes’ particulate matter was collected on each Cambridge filter. Two parallel filters were placed in a conical flask, and 10 mL of methyl tert-butyl ether (MTBE) and 100 μL of mixed standard solution (4 μg/mL methyl valerate, methyl heptanoate, methyl nonanoate, methyl undecanoate, and methyl tridecanoate dissolved in methanol) were added. After sealing and shaking for 30 minutes, the upper clear liquid was transferred to a chromatographic bottle for analysis.

Analysis Conditions:

One-dimensional column: DB-5MS chromatographic column, constant flow rate of 1.9 mL/min; two-dimensional column: DB-WAX chromatographic column, constant flow rate of 1.9 mL/min; injection port temperature: 250 °C; injection volume: 3 μL; injection mode: splitless injection; splitless time: 1 min; purge flow rate: 50 mL/min; heart-cutting time: cut 1 (5.1~10.0 min), cut 2 (10.0~16.6 min), cut 3 (16.6~23.5 min), cut 4 (23.5~30.5 min). One-dimensional temperature program: the initial temperature for all four cuts is 45 °C (held for 2 min), and then it is raised at a rate of 6 °C/min, reaching 93 °C for cut 1, 132.6 °C for cut 2, 174 °C for cut 3, and 216 °C for cut 4, followed by a rapid cooling to 60 °C (for cuts 1 and 2) or 80 °C (for cuts 3 and 4). Two-dimensional temperature program: cut 1 is raised at a rate of 4 °C/min to 180 °C, and then at a rate of 10 °C/min to 230 °C (20 min); cuts 2 and 3 are raised at a rate of 4 °C/min to 230 °C (20 min); cut 4 is raised at a rate of 4 °C/min to 230 °C (30 min). GC/MS interface temperature: 240 °C; electron energy: 70 eV; EI source temperature: 230 °C; quadrupole temperature: 150 °C; mass scanning range: 33~400 amu; peak area integration was performed using the selected ion monitoring method.

### 2.4 Sensory quality evaluation of heated tobacco products

The sensory evaluation indicators include 9 items: aroma quality, aroma concentration, offensive odor, strength, irritancy, dry sensation, bitterness, residual and stability. The Shanghai New Tobacco Research Institute Co., LTD organized 15 experts (with at least 10 years of experience in the tobacco field and all holding traditional cigarette sensory evaluation certificates. Before the evaluation, experts have received specialized training) to evaluate heated tobacco products. During the evaluation process, the experts puff the standard samples first, before the formal evaluation, the standard samples were first evaluated in a blind manner. The sensory evaluation criteria for heated tobacco products raw materials are shown in **Table 2** Each index is quantitatively scored. Evaluation samples were presented randomly, with evaluations conducted at 15-minute intervals. The total score for each individual index is 9 points, and the minimum scoring unit for each index is 0.5 points. The total score of the evaluation is the sum of all 7 indicators after removing strength and stability (the total score is 63 points). The sensory evaluation criteria for heated tobacco products can be found in reference (Fang et al., 2024).

**Table 2:** Sensory evaluation standards of heated tobacco products.

Score	Aroma quality	Aroma concentration	Offensive odor	Strength	Irritancy	Dry sensation	Bitterness	Residual	Stability
7~9	Favorable to Fine	Substantial to Abundant	Absent to Negligible	Pronounced to Intense	Non-irritating to Imperceptibly Irritating	Negligible to Slight	Undetectable to Faint	Residue-free & Smooth to Crystalline & Satisfying	Moderate to High Stability
4~6	Acceptable to Fair	Discernible to Moderate	Borderline to Appreciable	Moderate to Moderately Elevated	Minimally Perceptible to Discernible	Perceptible to Distinct	Perceptible to Distinct	Faintly Noticeable & Accommodating to Adequately Cleansed & Balanced	Marginally Stable to Acceptable
1~3	Deficient to Mediocre	Faint to Trace	Enough to Plenty	Faint to Subtle	Pronounced to Pungent	Pronounced to Pungent	Pronounced to Pungent	Harsh Afterfeel & Unpleasant to Aggressively Coating & Rejectable	Unstable to Borderline Stable

### 2.5 Data processing and statistical analysis

The raw data of the experiment was organized by Microsoft Excel 2021. IBM SPSS Statistics 24 was applied for canonical correlation analysis, Pearson correlation for correlation analysis, and ANOVA one-way analysis of variance for significant difference analysis, Pearson correlation for correlation analysis, and ANOVA one-way analysis and LSD of variance for significant difference analysis, based on the skewness and kurtosis, it can be concluded that each group of data is approximately normally distributed (skewness<2, kurtosis<7) (Curran et al., 1996), Intraclass Correlation Coefficient (ICC, two-way random, absolute agreement) for sensory Indicators score analysis. SIMCA 14.1 software was used for orthogonal partial least squares discriminant analysis (OPLS-DA), permutation test, and calculation of variable importance in projection (VIP). Cluster heatmap was performed using the Metware Cloud. Origin 2024 was used to create the component stacked bar chart and sensory score radar chart. The raw data of aroma components were Z-score standardized before subsequent analyses.

Components identification followed the standards of the Metabolomics Standards Initiative (MSI). Organic acids and neutral aroma components analyzed by GC–FID and LC-GC-MS, and smoke components analyzed by GC -MS, were identified by mass spectrum with the NIST 20.L (match factor ≥ 80%). Carotenoids and polyphenolic components, quantification was carried out using standard sample without mass spectrometric confirmation. Therefore, components all corresponds to MSI Level 2.

## 3 Results

### 3.1 Analysis of tobacco leaf powder aroma components

**Figure 1:**
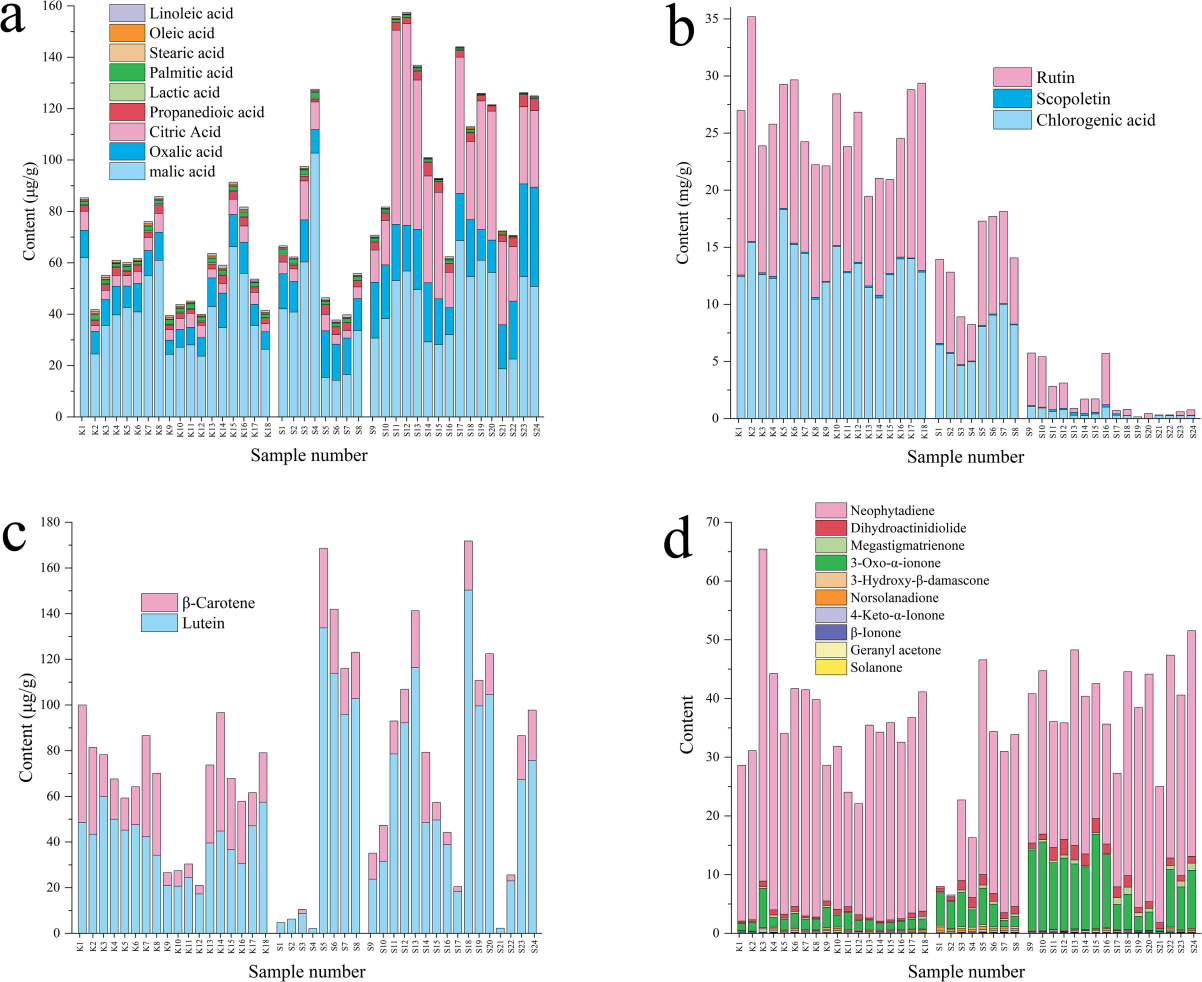
Stacked bar chart of aroma components in different tobacco leaf powders. **(a)** Non-volatile organic acids content; **(b)** Polyphenolic components content; **(c)** Carotenoid content; **(d)** Neutral fragrance components content.

A total of 24 powder aroma components were detected in different tobacco leaf samples, including 9 non-volatile organic acids, 3 polyphenols, 2 carotenoids and 10 neutral flavoring components. The specific content analysis is shown in **Figure 1**. There are significant differences in the content of aroma components in the powder of different tobacco leaf samples.

#### 3.1.1 Analysis of non-volatile organic acid content

Non-volatile organic acids generally refer to organic acids with carbon atom numbers ranging from 2 to 6. They can combine with alkaloids to regulate the content of free and protonated nicotine in tobacco, thereby adjusting the pH of the smoke and greatly influencing the strength and taste (Li DK. et al., 2021). The content of citric acid (especially S11, S12) and oxalic acid (especially S23, S24) in sun-cured red tobacco is higher, but the contents of oleic acid, stearic acid and lactic acid are all lower. Flue-cured tobacco and sun-cured yellow tobacco have higher contents of palmitic acid and linoleic acid.

#### 3.1.2 Analysis of phenols component content

Phenols have a significant impact on the aroma of cigarettes. Studies have shown that chlorogenic acid and rutin both have a positive effect on the aroma quality, and their partial correlation coefficients both reach extremely significant levels. However, scopoletin has a negative impact (Jiang et al., 2014). **Figure 1b** shows that the content of phenols in flue-cured tobacco is the highest, followed by sun-cured yellow tobacco, and the least in sun-cured red tobacco. The content of phenols in some samples is close to 0, such as S19, S20, S21 and S22. Among the 3 phenols, the content of scopoletin is very low.

#### 3.1.3 Analysis of carotenoid components

Carotenoids are precursors to many aroma components, and many derivative components significantly contribute to cigarette aroma (Shi et al., 2023). The overall content of carotenoids (Li et al., 2022) in flue-cured tobacco is lower, but the content of β-carotene is higher (but K9, K10, K11 and K12 still had a low content of β-carotene). The content of β-carotene in sun-cured yellow and sun-cured red tobacco is lower, S1, S2, S4 and S21 almost zero, but the content of lutein is higher, especially S5, S6, S13 and S18.

#### 3.1.4 Analysis of neutral aroma components

The content of neutral aroma components has a significant correlation with the aroma quality of cigarettes. Neophytadiene is the most abundant neutral aroma component, but the content of neophytadiene in S1 and S2 samples is very low. The contents of 3-oxo-α-violet alcohol, megastigmatrienone and dihydroactinidiolide in sun-cured yellow tobacco and sun-cured red tobacco are very high. The content of 3-hydroxyl-β-damascone and solandione in sun-cured yellow tobacco are relatively high.

### 3.2 Analysis of pyrolysis components in tobacco leaves

**Figure 2:**
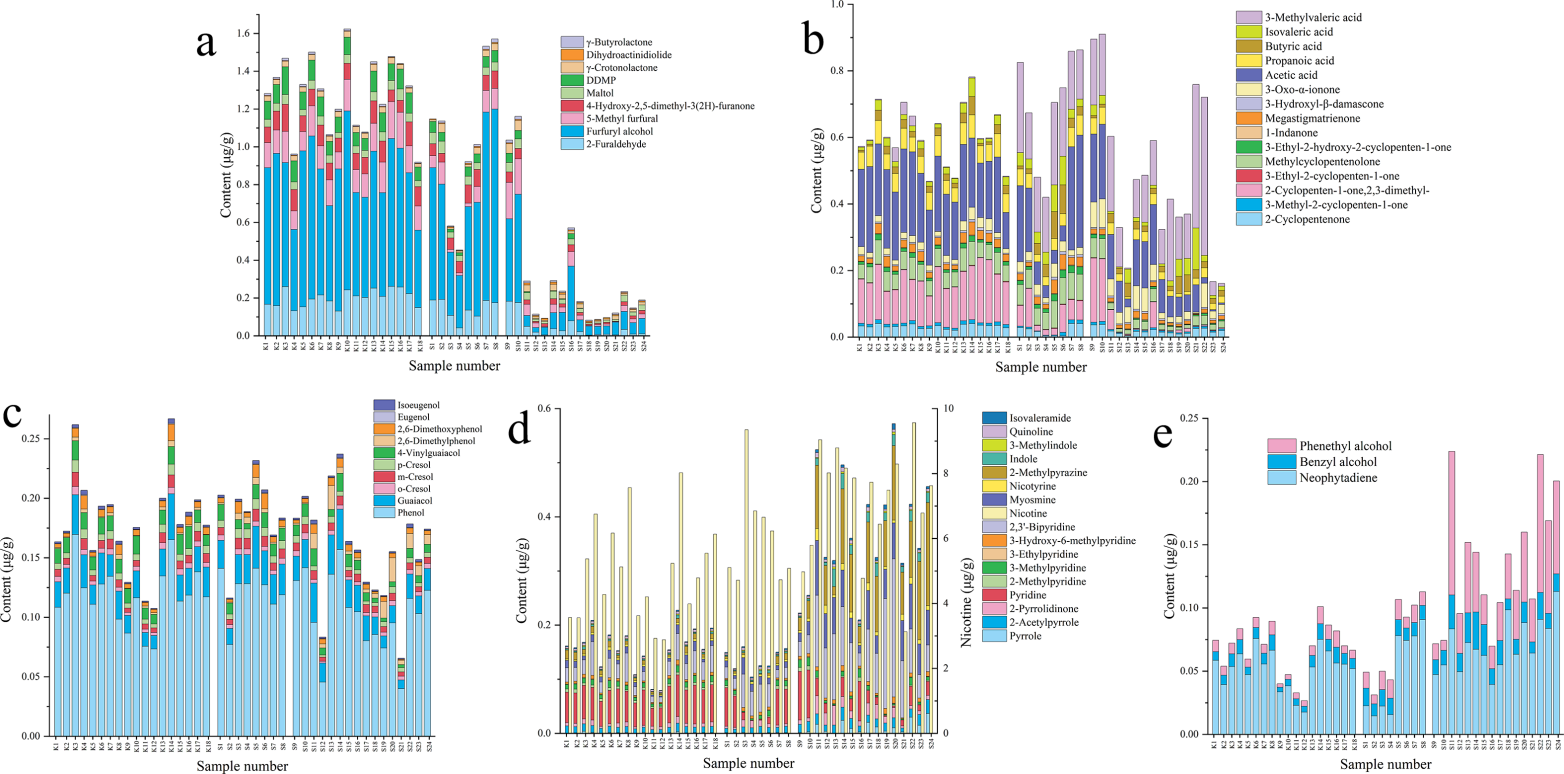
Stacked bar chart of heated tobacco smoke components in different tobacco leaves. **(a)** Pyran, furan and lactones; **(b)** Ketones and organic acids; **(c)** Phenols; **(d)** Nitrogen-containing components; **(e)** Other components.

A total of 54 smoke components were detected in different tobacco leaf samples, including 9 pyran, furan and lactones; 15 ketones and organic acids; 10 phenols; 17 nitrogen-containing components and 3 other components. The specific content analysis is shown in **Figure 2**. There are significant differences in the content of smoke components of different tobacco leaf samples.

#### 3.2.1 Analysis of pyran, furan and lactones components

The content of pyran, furan and lactones components in some sun-cured red tobacco is extremely low, but that in flue-cured tobacco is the highest. The content varies greatly among different samples of sun-cured yellow tobacco, but the proportion of furfuryl alcohol in the 3 types of tobacco leaves is the highest.

#### 3.2.2 Analysis of ketones and organic acids components

Among the 3 types of tobacco leaves, the contents of 3-methyl-2-cyclopenten-1-one, 3-ethyl-2-cyclopenten-1-one, 1-indanone and 3-oxo-α-violet alcohol are all very low. The contents of ketones and organic acids in some sun-cured red tobacco are relatively low, and the contents varies greatly among the samples. The content of 2-cyclopenten-1-one,2,3-dimethyl-, 2-cyclopentenone, methylcyclopentenolone, megastigmatrienone, acetic acid, propanoic acid in flue-cured tobacco is relatively high, but the content of 3-methylvaleric acid is extremely low. The contents of methylcyclopentenolone, acetic acid, isovaleric acid and 3-methylvaleric acid in sun-cured yellow tobacco are relatively high, but the content of 2-cyclopenten-1-one,2,3-dimethyl- is relatively low. The content of acetic acid, isovaleric acid and 3-methylvaleric acid in sun-cured red tobacco is relatively high, but the content of other substances is relatively low.

#### 3.2.3 Analysis of phenolic substances

The phenolic substances content of samples K10, K11, S2, S12 and S21 is relatively low, while the proportion of phenol content in the 3 types of tobacco leaves is the highest. The content of 2, 6-dimethoxyphenol is relatively high in flue-cured tobacco and sun-cured yellow tobacco, while the content of 2, 6-dimethylphenol is relatively high in sun-cured red tobacco.

#### 3.2.4 Analysis of nitrogen-containing components

The overall nitrogen-containing components in flue-cured tobacco and sun-cured yellow tobacco are relatively low, while in sun-cured red tobacco are relatively high. The contents of pyrrole, 2-pyrrolidinone, 2,3’-bipyridine, myosmine, nicotyrine, 2-methylpyrazine, indole, quinoline and isovaleramide are all higher than those in flue-cured tobacco and sun-cured yellow tobacco, and the content of 2-methylpyrazine is extremely high. Among the 3 types of tobacco leaves, the nicotine content is the highest.

### 3.3 Screening of key tobacco leaf powder aroma components

**Figure 3:**
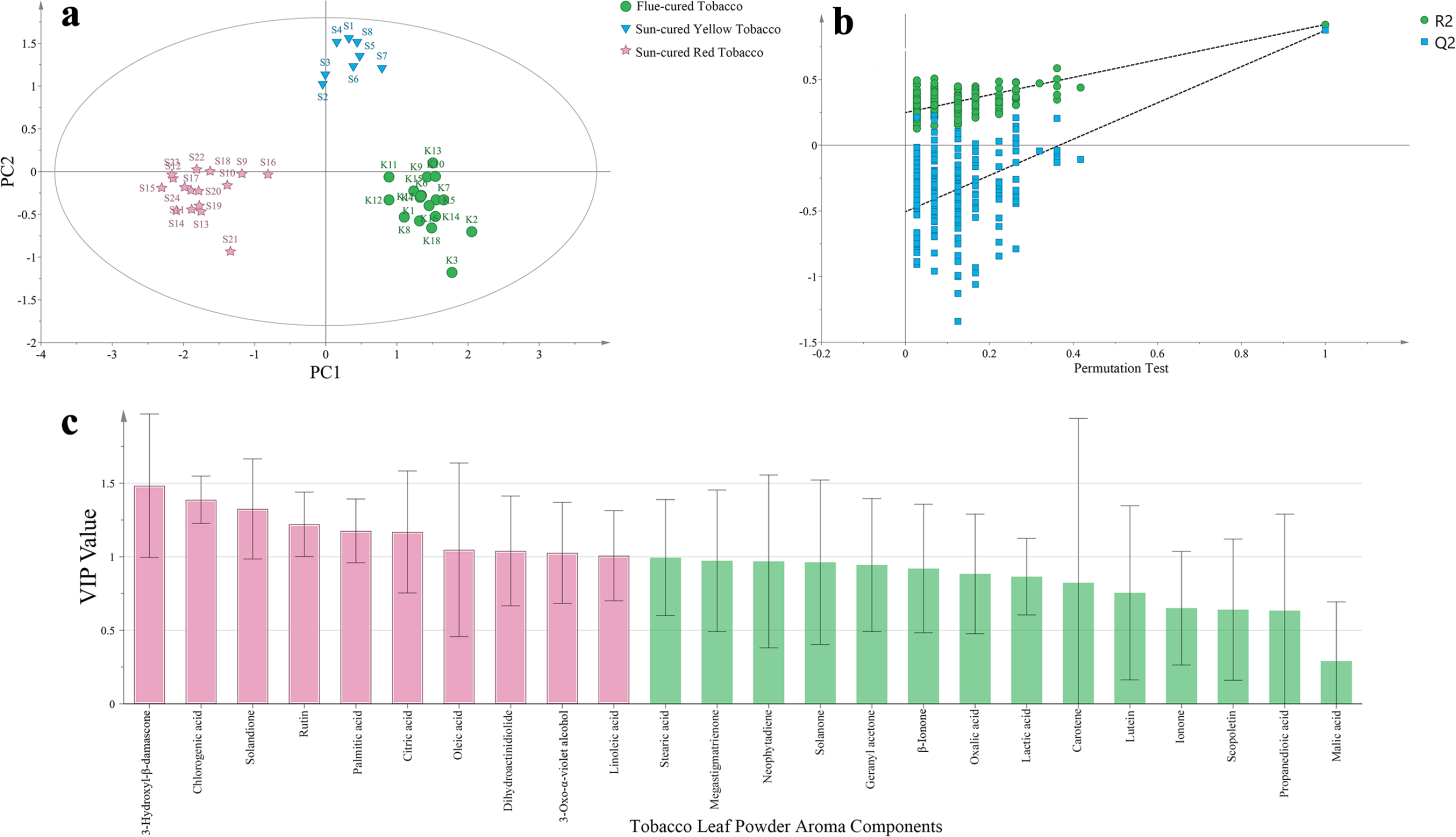
**(a)** OPLS-DA score chart of tobacco leaf powder aroma components, **(b)** substitution verification model, **(c)** VIP value.

Taking 24 kinds of tobacco leaf powder aroma components as dependent variables, and different types of tobacco leaves (flue-cured tobacco, sun-cured yellow tobacco and sun-cured red tobacco) as independent variables, the effective differentiation of 3 different types of tobacco leaf samples can be achieved through OPLS-DA (**Figure 3a**). In this analysis, the fitting index of the independent variable (R^2^X) is 0.659, the fitting index of the dependent variable (R^2^Y) is 0.913, and the model prediction index (Q^2^) is 0.87. If R^2^ and Q^2^ exceed 0.5, it indicates that the model fitting results are acceptable (Yun et al., 2021). After 200 permutation tests, as shown in **Figure 3b**, the intersection point of the regression line of Q^2^ and the vertical axis is less than 0, indicating that there is no overfitting in this model and the model is verified to be valid.

Based on the criteria of P<0.05 and VIP>1, 10 tobacco leaf powder aroma components were screened out, namely 3-hydroxyl-β-damascone, chlorogenic acid, solandione, rutin, palmitic acid, citric acid, oleic acid, dihydroactinidiolide, 3-oxo-α-violet alcohol and linoleic acid, as shown in **Figure 3c**. There are 4 non-volatile organic acids, 2 polyphenols and 4 neutral aroma components.

### 3.4 Correlation analysis between key aroma components and sensory quality of tobacco leaf powder

Pearson correlation analysis was conducted on the 10 key aroma components obtained, as shown in **Table 3**. The aroma components of tobacco leaf powder were highly significantly correlated with the sensory quality of heated tobacco products, including aroma quality, irritancy, dry sensation, bitterness, residual and total score. Among them, citric acid, 3-oxo-α-violet alcohol and dihydroactinidiolide were significantly negatively correlated with the above sensory indicators, while palmitic acid, oleic acid, linoleic acid, chlorogenic acid and rutin were significantly positively correlated with the above sensory indicators. This indicates that the lower the content of citric acid, 3-oxo-α-violet alcohol and dihydroactinidiolide, and the higher the content of palmitic acid, oleic acid, linoleic acid, chlorogenic acid and rutin, were statistically associated with the better aroma quality of heated tobacco, the smaller irritancy, dry sensation, bitterness and residual perception, the more comfortable smoking experience, and the higher total score.

**Table 3:** Pearson correlation analysis results between key aroma components in tobacco leaf powder and heated tobacco product sensory scores (df = 40).

Components	Aroma quality	Aroma concentration	Offensive odor	Strength	Irritancy	Dry sensation	Bitterness	Residual	Stability	Total score
Citric acid	-0.341*	0.132	-0.299	0.141	-0.609**	-0.613**	-0.689**	-0.677**	-0.189	-0.672**
Palmitic acid	0.218	-0.171	0.195	0.015	0.385*	0.532**	0.539**	0.548**	0.118	0.497**
Oleic acid	0.325*	-0.156	0.14	0.141	0.355*	0.403**	0.426**	0.464**	0.012	0.430**
Linoleic acid	0.283	-0.038	0.162	0.247	0.349*	0.417**	0.508**	0.472**	0.183	0.460**
Chlorogenic acid	0.377*	-0.173	0.269	0.076	0.489**	0.526**	0.573**	0.650**	0.129	0.573**
Rutin	0.394**	-0.137	0.273	0.003	0.485**	0.451**	0.535**	0.612**	0.073	0.535**
Solandione	0.018	0.132	0.216	-0.062	0.008	0.184	0.238	0.114	0.053	0.177
3-Hydroxyl-β-damascone	-0.039	0.282	0.388*	-0.132	0.017	0.173	0.178	-0.046	0.069	0.199
3-Oxo-α-violet alcohol	-0.419**	0.041	-0.182	-0.112	-0.437**	-0.392*	-0.386*	-0.539**	-0.200	-0.476**
Dihydroactinidiolide	-0.400**	0.232	0.000	0.044	-0.715**	-0.641**	-0.544**	-0.775**	-0.312*	-0.623**

**means *p* < 0.01. *means *p* < 0.05.

To further elucidate overall relationships, CCA was performed using the 10 key aroma constituents (x1–x10) as set X and 10 sensory attributes (y1–y10) as set Y (**Table 4**), In the correlation analysis between set X and set Y, only the correlation coefficients of the first pair of canonical correlation variables reached a significant level (P<0.05), with a correlation coefficient of r=0.877, so this canonical correlation variable can represent the overall correlation well between the aroma components of these 10 tobacco leaf powders and sensory quality.

**Table 4:** Canonical correlation analysis between key tobacco leaf powder aroma components and sensory evaluation indicators of heated tobacco products.

Set	Correlation	Characteristic value	Wilk’s	*F*	*P*	Canonical redundancy analysis			
						X×Itself	X×Y	Y×Itself	Y×X
1	0.877	3.320	0.013	1.417	0.023	0.428	0.329	0.372	0.286
2	0.791	1.667	0.056	1.097	0.309	0.153	0.096	0.038	0.024
3	0.668	0.806	0.148	0.888	0.701	0.064	0.028	0.050	0.022
4	0.592	0.540	0.268	0.794	0.820	0.068	0.024	0.141	0.050
5	0.542	0.416	0.412	0.726	0.865	0.053	0.016	0.080	0.024
6	0.519	0.369	0.584	0.635	0.904	0.038	0.010	0.094	0.025
7	0.350	0.139	0.799	0.409	0.977	0.051	0.006	0.034	0.004
8	0.253	0.068	0.911	0.308	0.970	0.054	0.003	0.085	0.005
9	0.163	0.027	0.973	0.206	0.934	0.052	0.001	0.057	0.002
10	0.019	0.000	1.000	0.011	0.917	0.039	0.000	0.049	0.000

The canonical correlation coefficient represents the importance of the original variable in the extracted canonical variable expression (Zhou et al., 2024). Due to the differences in measurement units of the original variables, so used the standardized canonical coefficients, as shown in **Table 5**.

**Table 5:** The canonical correlation coefficient between the tobacco leaf powder aroma components and sensory evaluation indicators (include 10 aroma components and 10 sensory indicators).

Aroma components	Canonical correlation coefficient	Sensory evaluation indicators	Canonical correlation coefficient
3-Hydroxyl-β-damascone x_1_	-0.014	Aroma quality y_1_	-0.101
Chlorogenic acid x_2_	0.274	Aroma concentration y_2_	0.198
Solandione x_3_	-0.113	Offensive odor y_3_	0.553
Rutin x_4_	-0.089	Strength y_4_	0.235
Palmitic acid x_5_	0.474	Irritancy y_5_	0.423
Citric acid x_6_	0.008	Dry sensation y_6_	0.611
Oleic acid x_7_	-0.075	Bitterness y_7_	-0.178
Dihydroactinidiolide x_8_	-0.759	Residual y_8_	1.405
3-Oxo-α-violet alcohol x_9_	0.058	Stability y_9_	-0.013
Linoleic acid x_10_	-0.126	Total score y_10_	-1.250

The canonical correlation equation is as follows:


$$\begin{array}{c} \text{U}=-0.014{\text{x}}_{1}+0.274{\text{x}}_{2}-0.113{\text{x}}_{3}-0.089{\text{x}}_{4}+0.474{\text{x}}_{5}+0.008{\text{x}}_{6}\\ -0.075{\text{x}}_{7}-0.759{\text{x}}_{8}+0.058{\text{x}}_{9}-0.126{\text{x}}_{10} \end{array}$$

$$\begin{array}{c} \text{V}=-0.101{\text{y}}_{1}+0.198{\text{y}}_{2}+0.553{\text{y}}_{3}+0.235{\text{y}}_{4}+0.423{\text{y}}_{5}+0.611{\text{y}}_{6}\\ -0.178{\text{y}}_{7}+1.405{\text{y}}_{8}-0.013{\text{y}}_{9}-1.250{\text{y}}_{10} \end{array}$$

Through canonical redundancy analysis, it can be known that the canonical variables in set X can explain 42.8% of the total variation of tobacco leaf powder aroma components, and the canonical variables in set Y can explain 37.2% of the total variation of sensory evaluation indicators.

Standardized canonical coefficients (**Table 5**) indicated that chlorogenic acid (x2), palmitic acid (x5), and dihydroactiniolide (x8) are relatively large, with canonical loads of 0.274, 0.474, and -0.759 respectively; In the canonical variable Y, the loads of offensive odor (y3), irritancy (y5), dry sensation (y6), residual (y8), and total score (y10) are relatively high, with canonical loads of 0.553, 0.423, 0.611, 1.405, and -1.250 respectively. It illustrates that this canonical variable is mainly determined by this substances and indicators. When the contents of palmitic acid and chlorogenic acid in the tobacco powder are relatively high, while the content of dihydroactiniolide is low, the total score of heated tobacco could be higher, and the negative indicators such as residual, dry sensation, offensive odor and irritancy could be significantly reduced.

### 3.5 Screening of key smoke components in heated tobacco products

**Figure 4:**
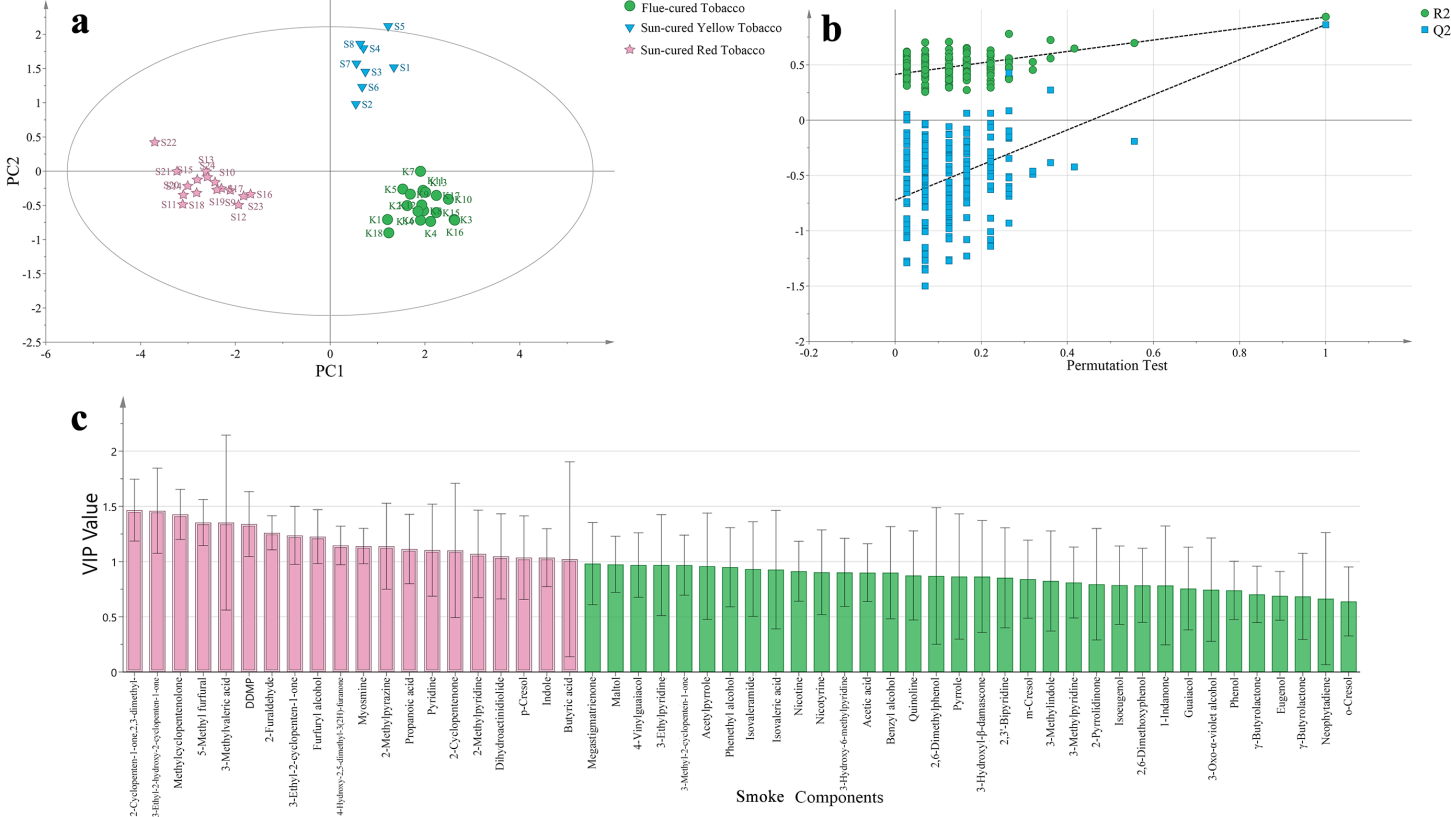
**(a)** OPLS-DA score chart of heated tobacco smoke components, **(b)** substitution verification model, **(c)** VIP value.

Taking 54 components of heated tobacco products as dependent variables, and different types of tobacco leaves as independent variables, the effective differentiation of 3 different types of tobacco leaf samples can be achieved through OPLS-DA (**Figure 4a**). In this analysis, the fitting index of the independent variable (R2X) is 0.818, the fitting index of the dependent variable (R2Y) is 0.935, and the model prediction index (Q2) is 0.799. If R2 and Q2 exceed 0.5, it indicates that the model fitting result is acceptable (Yun et al., 2021). After 200 permutation tests, as shown in **Figure 4b**, the intersection point of the regression line of Q2 and the vertical axis is less than 0, indicating that there is no overfitting in this model and the model is verified to be valid.

Based on the criteria of P<0.05 and VIP>1, 2-furaldehyde, furfuryl alcohol, 5-methyl furfural, dihydroactinidiolide, p-cresol, 2-cyclopenten-1-one,2,3-dimethyl-, 3-ethyl-2-hydroxy-2-cyclopenten-1-one, methylcyclopentenolone, 3-methylvaleric acid, pyridine, 2-methylpyridine and 2-methylpyrazine were screened out. There are 12 components of heated tobacco products, as shown in **Figure 4c**. There are 4 furans, pyrans and lactones; 1 phenol; 4 ketones and organic acids; and 3 nitrogen-containing components.

### 3.6 Correlation analysis between key smoke components and sensory quality of heated tobacco products

Pearson correlation analysis was conducted on the 20 key smoke components obtained, as shown in **Table 6**. The key components of the smoke from heated tobacco are highly significantly correlated with the irritancy, dry sensation, bitterness, residual and total scores of the sensory quality of heated tobacco. Among them, dihydroactinidiolide, 3-methylvaleric acid and 2-methylpyrazine are significantly negatively correlated with the above sensory indicators, while 2-furaldehyde, furfuryl alcohol, 5-methyl furfural, 2-cyclopenten-1-one, 2,3-dimethyl-, 3-ethyl-2-hydroxy-2-cyclopenten-1-one, methylcyclopentenolon, pyridine and 2-methylpyridine are significantly positively correlated with the above sensory indicators. This indicates that the higher the content of these components, the lower the content of dihydroactinidiolide and 3-methylvaleric acid, and the lower the irritancy, dry sensation, bitterness and residual sensation of heated tobacco, and the more comfortable smoking experience, and the higher total score.

**Table 6:** Pearson correlation analysis results between key mainstream smoke aroma components of heated tobacco and product sensory quality scores (df=40).

Components	Aroma quality	Aroma concentration	Offensive odor	Strength	Irritancy	Dry sensation	Bitterness	Residual	Stability	Total score
2-Furaldehyde	0.297	-0.255	0.186	-0.111	0.626**	0.698**	0.719**	0.718**	0.177	0.652**
Furfuryl alcohol	0.273	-0.203	0.249	-0.116	0.583**	0.633**	0.643**	0.645**	0.212	0.609**
5-Methyl furfural	0.304	-0.25	0.156	-0.129	0.627**	0.648**	0.656**	0.674**	0.166	0.622**
Dihydroactinidiolide	-0.296	0.109	-0.244	0.125	-0.570**	-0.513**	-0.454**	-0.595**	-0.163	-0.554**
p-Cresol	0.314*	0.251	0.236	0.159	0.214	0.207	0.419**	0.316*	0.196	0.396**
2-Cyclopenten-1-one,2,3-dimethyl-	0.289	-0.246	0.127	-0.125	0.598**	0.594**	0.606**	0.653**	0.152	0.576**
Methylcyclopentenolone	0.239	0.012	0.307*	-0.121	0.501**	0.506**	0.658**	0.524**	0.263	0.600**
3-Ethyl-2-hydroxy-2-cyclopenten-1-one	0.231	0.139	0.325*	-0.037	0.408**	0.357*	0.554**	0.390*	0.281	0.516**
3-Methylvaleric acid	-0.233	0.265	-0.033	-0.229	-0.221	-0.241	-0.183	-0.381*	-0.005	-0.217
Pyridine	0.225	-0.245	0.088	-0.2	0.557**	0.628**	0.639**	0.627**	0.122	0.577**
2-Methylpyridine	0.234	-0.211	0.121	-0.174	0.569**	0.542**	0.536**	0.555**	0.16	0.530**
2-Methylpyrazine	-0.065	0.199	-0.225	0.121	-0.378*	-0.398**	-0.484**	-0.499**	-0.032	-0.409**

**means *p* < 0.01. *means *p* < 0.05.

CCA was further conducted with the 12 key aerosol components (x11–x22) as set X and the 10 sensory attributes (y1–y10) as set Y (**Table 7**). In the correlation analysis between set X and set Y, only the correlation coefficients of the first pair of canonical correlation variables reached a significant level (P<0.05), with a correlation coefficient of r=0.924, so this canonical correlation variable can represent the overall correlation well between the 12 heated tobacco smoke components and sensory quality.

**Table 7:** Canonical correlation analysis between key heated tobacco smoke components and sensory evaluation indicators of heated tobacco products.

Set	Correlation	Characteristic value	Wilk’s	*F*	*P*	Canonical redundancy analysis			
						X×Itself	X×Y	Y×Itself	Y×X
1	0.924	5.807	0.005	1.377	0.028	0.346	0.295	0.231	0.197
2	0.768	1.436	0.035	0.981	0.537	0.174	0.102	0.156	0.092
3	0.734	1.166	0.085	0.878	0.738	0.099	0.053	0.120	0.065
4	0.646	0.716	0.185	0.753	0.896	0.059	0.025	0.049	0.020
5	0.612	0.600	0.317	0.670	0.942	0.045	0.017	0.050	0.019
6	0.505	0.342	0.507	0.539	0.981	0.037	0.009	0.141	0.036
7	0.428	0.225	0.680	0.447	0.986	0.037	0.007	0.026	0.005
8	0.343	0.133	0.833	0.342	0.988	0.081	0.009	0.070	0.008
9	0.221	0.051	0.944	0.204	0.989	0.046	0.002	0.046	0.002
10	0.085	0.007	0.993	0.070	0.975	0.026	0.000	0.111	0.001

The canonical correlation coefficients are shown in **Table 8**.

**Table 8:** The canonical correlation coefficient between the key heated tobacco smoke components and sensory evaluation indicators (include 12 smoke components and 10 sensory indicators).

Heated tobacco smoke components	Canonical correlation coefficient	Sensory evaluation indicators	Canonical correlation coefficient
2-Furaldehyde x_11_	-0.626	Aroma quality y_1_	0.167
Furfuryl alcohol x_12_	-0.522	Aroma concentration y_2_	-0.512
5-Methyl furfural x_13_	-1.764	Offensive odor y_3_	-0.855
Dihydroactinidiolide x_14_	-0.024	Strength y_4_	-0.219
p-Cresol x_15_	-0.330	Irritancy y_5_	-0.115
2-Cyclopenten-1-one,2,3-dimethyl- x_16_	2.331	Dry sensation y_6_	-1.600
Methylcyclopentenolone x_17_	-1.426	Bitterness y_7_	-0.817
3-Ethyl-2-hydroxy-2-cyclopenten-1-one x_18_	2.149	Residual y_8_	-1.572
3-Methylvaleric acid x_19_	0.032	Stability y_9_	0.170
Pyridine x_20_	-0.128	Total score y_10_	3.163
2-Methylpyridine x_21_	0.156		
2-Methylpyrazine x_22_	0.399		

The canonical correlation equation is as follows:


$$\text{U}=-0.626{\text{x}}_{11}-0.522{\text{x}}_{12}-1.764{\text{x}}_{13}-0.024{\text{x}}_{14}-0.330{\text{x}}_{15}+2.331{\text{x}}_{16}-1.426{\text{x}}_{17}+2.149{\text{x}}_{18}+0.032{\text{x}}_{19}-0.128{\text{x}}_{20}+0.156{\text{x}}_{21}+0.399{\text{x}}_{22}$$

$$\text{V}=0.167{\text{y}}_{1}-0.512{\text{y}}_{2}-0.855{\text{y}}_{3}-0.219{\text{y}}_{4}-0.115{\text{y}}_{5}-1.600{\text{y}}_{6}-0.817{\text{y}}_{7}-1.572{\text{y}}_{8}+0.170{\text{y}}_{9}+3.163{\text{y}}_{10}$$

Through canonical redundancy analysis, it can be known that the canonical variables in set X can explain 34.6% of the total variation of heated tobacco smoke components, and the canonical variables in set Y can explain 23.1% of the total variation of sensory evaluation indicators.

Standardized canonical coefficients (**Table 8**) revealed that 2-furaldehyde (x11), furfuryl alcohol (x12), 5-methyl furfural (x13), 2-cyclopenten-1-one,2,3-dimethyl- (x16), methylcyclopentenolone (x17), and 3-ethyl-2-hydroxy-2-cyclopenten-1-one (x18) are relatively large, with canonical loads of -0.626, -0.522, -1.764, 2.331, -1.426 and 2.149 respectively; In the canonical variable Y, the loads of aroma concentration (y2), offensive odor (y3), dry sensation (y6), bitterness (y7), residual (y8), and total score (y10) are relatively high, with canonical loads of -0.512, -0.855, -1.600, -0.817, -1.572, and 3.163 respectively. It illustrates that this canonical variable is mainly determined by this substances and indicators. When the contents of 2-cyclopenten-1-one,2,3-dimethyl- and 3-ethyl-2-hydroxy-2-cyclopenten-1-one in the smoke components are relatively high, while the contents of 2-furaldehyde, furfuryl alcohol, 5-methyl furfural and methylcyclopentenol are relatively low, the total score of heated tobacco could be higher, and the negative indicators such as residual, dry sensation, offensive odor and bitterness could be significantly reduced.

### 3.7 Cluster heatmap analysis of key aroma components

**Figure 5:**
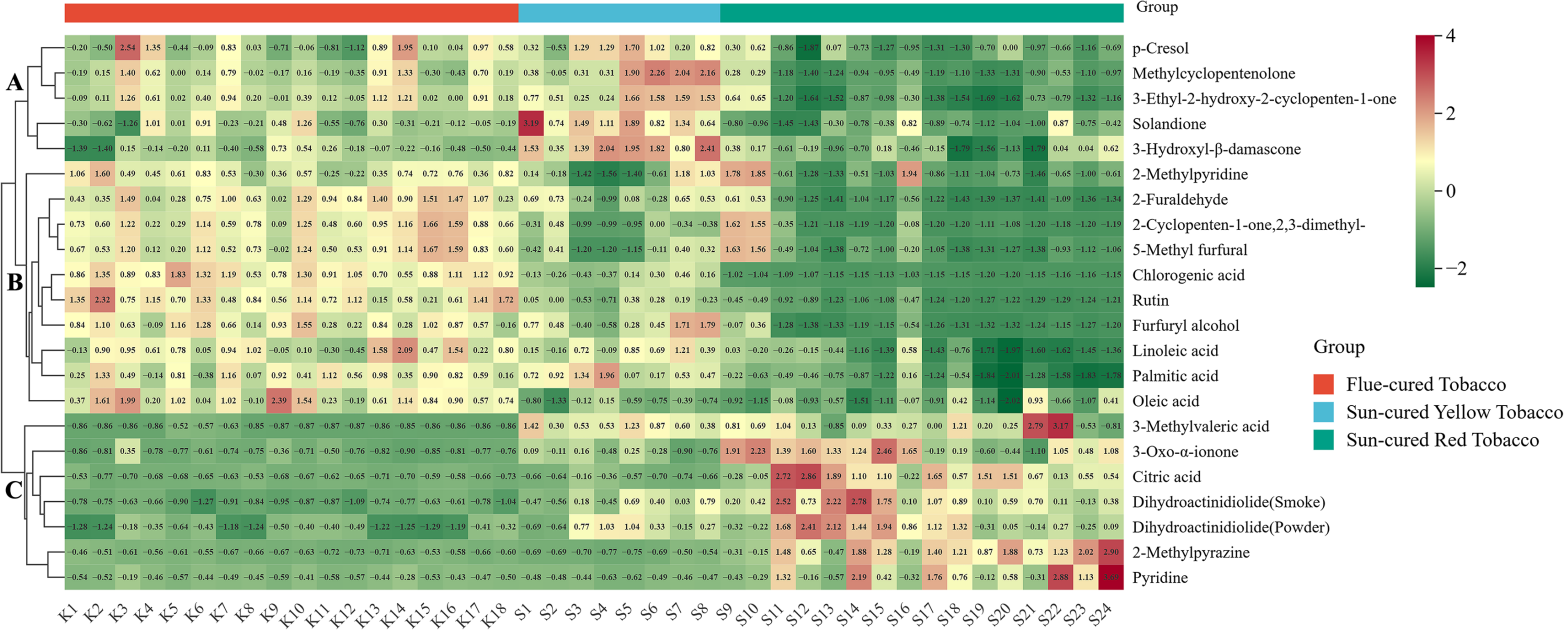
Cluster heatmap of key aroma components.

To further analyze the impact of differences in key aroma components on different types of tobacco, the selected key components were visualized, as shown in **Figure 5**. The 22 key aroma components can be classified into three categories: A, B, and C. Among them, category A includes 7 components, namely 3-methylvaleric acid, dihydroactinidiolide, citric acid, 3-oxo-α-violet alcohol, 2-methylpyrazine, and pyridine. The content of these components is the lowest in flue-cured tobacco and the highest in sun-cured red tobacco; category B includes 2-methylpyridine, oleic acid, 2-furaldehyde, 2-cyclopenten-1-one,2,3-dimethyl-, 5-methylfurfural, furfuryl alcohol, chlorogenic acid, rutin, linoleic acid, and palmitic acid. The content of these components is the lowest in sun-cured red tobacco and the highest in flue-cured tobacco; category C includes methylcyclopentenolone, 3-ethyl-2-hydroxy-2-cyclopenten-1-one, p-cresol, solandione, and 3-hydroxyl-β-damascone, which have the lowest content in sun-cured red tobacco and the highest in sun-cured yellow tobacco.

### 3.8 Analysis of sensory scores of heated tobacco products

**Figure 6:**
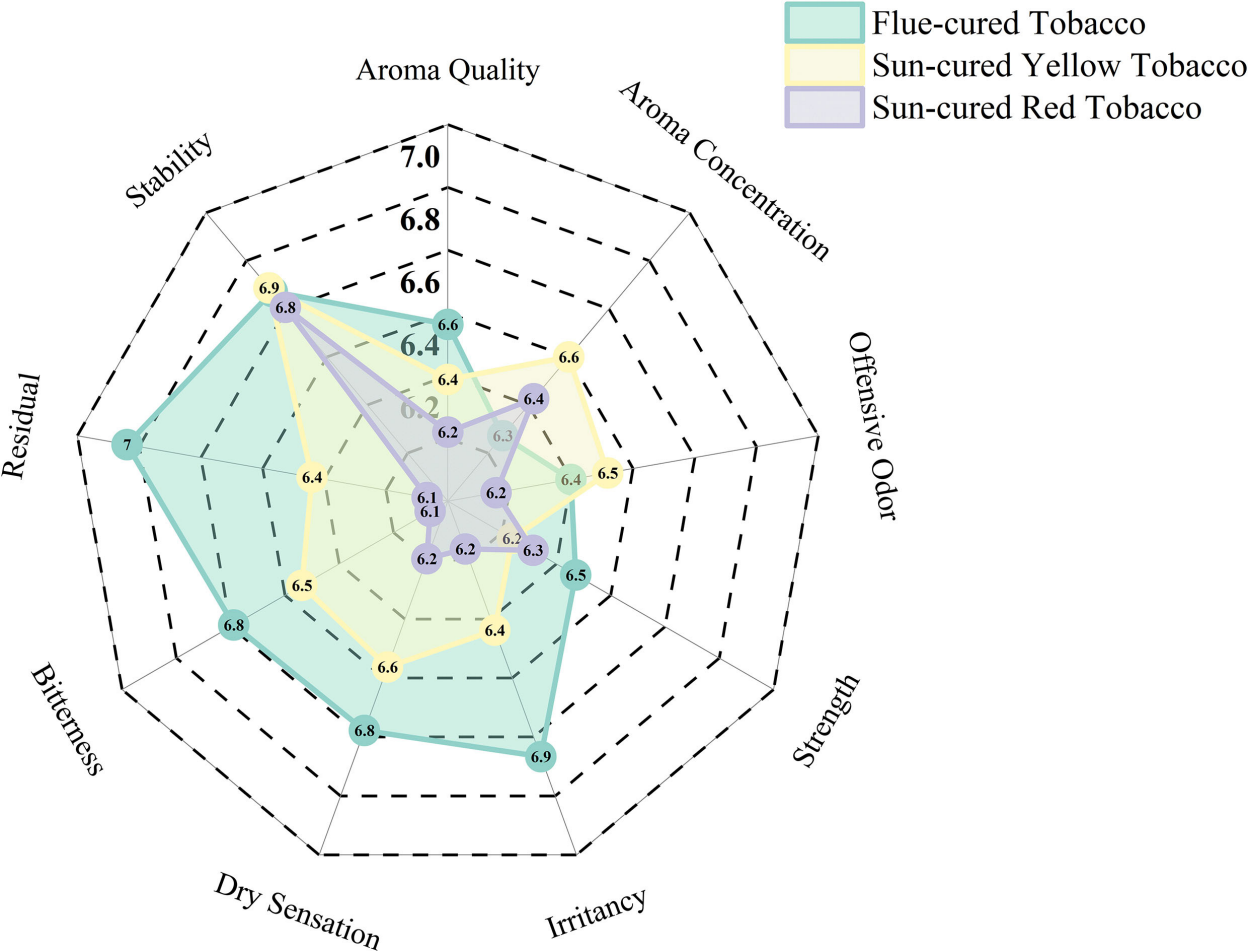
Sensory score radar chart of heated tobacco products.

As can be seen from the radar chart (**Figure 6**), the stability, irritancy, dry sensation, bitterness and residual scores of flue-cured tobacco are relatively high, but the aroma concentration score is relatively low. The stability, aroma concentration, offensive odor, dry sensation and bitterness scores of sun-cured yellow tobacco are relatively high, but the strength score is relatively low. The stability, aroma concentration and strength scores of sun-cured red tobacco are relatively high, but the aroma quality, offensive odor, bitterness and residual are relatively low.

Through analysis of variance (**Table 9**), it can be known that the scores of aroma quality and bitterness of flue-cured tobacco are significantly higher than those of sun-cured red tobacco, and the scores of irritancy, dry sensation and residual are significantly higher than those of sun-cured yellow tobacco and sun-cured red tobacco. However, except for the aroma quality, there was no significant difference in the scores of other indicators between sun-cured yellow tobacco and sun-cured red tobacco.

**Table 9:** ANOVA and ICC results of sensory quality scores of heated tobacco products prepared from different types of tobacco raw materials (df = 39).

Type	Aroma quality	Aroma concentration	Offensive odor	Strength	Irritancy	Dry sensation	Bitterness	Residual	Stability	Total score
Flue-cured Tobacco	6.56 ± 0.41a	6.27 ± 0.39a	6.40 ± 0.58a	6.47 ± 0.77a	6.87 ± 0.48a	6.78 ± 0.40a	6.79 ± 0.36a	7.04 ± 0.44a	6.87 ± 0.28a	46.46 ± 1.72a
Sun-cured Yellow Tobacco	6.39 ± 0.20ab	6.60 ± 0.52a	6.52 ± 0.32a	6.24 ± 0.86a	6.44 ± 0.57ab	6.56 ± 0.49ab	6.54 ± 0.40a	6.44 ± 0.43b	6.89 ± 0.10a	45.29 ± 1.78a
Sun-cured Red Tobacco	6.22 ± 0.33b	6.43 ± 0.31a	6.16 ± 0.30a	6.31 ± 0.58a	6.16 ± 0.63b	6.20 ± 0.39b	6.07 ± 0.55b	6.07 ± 0.47b	6.81 ± 0.15a	43.22 ± 2.41b
*F*	4.081	2.067	2.112	0.358	6.828	8.409	11.333	20.284	0.571	10.976
*P*	0.025	0.140	0.135	0.701	0.003	0.001	0.000	0.000	0.570	0.000
ICC (average measures	0.580	0.649	0.530	0.825	0.780	0.658	0.665	0.751	0.134	/

## 4 Discussion

### 4.1 The influence of tobacco leaf powder aroma components on sensory quality

The composition of aroma constituents differed markedly among different tobacco leaves. Flue-cured tobacco contained higher levels of polyphenols and neutral aroma components, especially chlorogenic acid, rutin and neophytadiene, consistent with previous studies (Zhou et al., 2025). Sun-cured yellow tobacco showed greater variability in aroma components, such as malic acid and carotene, whereas sun-cured red tobacco had a relatively high levels of non-volatile organic acids, carotenoids and neutral aroma components, especially citric acid, rutin, neophytadiene and 3-oxo-α-ionol.

Non-volatile organic acids can affect the taste of smoke by adjusting smoke pH and by regulating the content of protonated and free nicotine in the smoke, thus affecting irritancy (Chen et al., 2025). Among the 10 key leaf powder aroma components identified by OPLS-DA, the scores of irritancy, dry sensation and residual of flue-cured tobacco and sun-cured yellow tobacco with higher chlorogenic acid content were relatively high, so the heated tobacco products made from tobacco leaves with higher chlorogenic acid content had a higher score with smoothness, cleanliness and softness, consistent with earlier studies (Shen et al., 2021; Hao et al., 2022). Palmitic acid had a positive influence on aroma quality (r = 0.218), offensive odor (r = 0.195) and irritancy (r = 0.385), whereas oleic and linoleic acids had a negative effects on these indexes. These relationships can be used as criteria for raw material selection. Dihydroactinidiolide, a neutral aroma component, showed a fumi contribution to sensory quality, also in agreement with previous findings (Huang et al., 2024).

Taken together, the present results indicate that both the overall level and the balance of specific organic acids, polyphenols and neutral aroma constituents in leaf powder play an important role in HTP sensory quality.

### 4.2 The influence of heated tobacco smoke components on sensory quality

The heated tobacco smoke components had significant differences among three tobacco types. The smoke components from flue-cured and sun-cured yellow tobaccos were relatively similar, with higher contents of furan and pyran and lactone, phenols, ketone and organic acid, especially furfural (Banožić et al., 2020), furfuryl alcohol, 2-cyclopenten-1-one, 2,3-dimethyl-, acetic acid and pyridine, consistent with previous observations (Zhao et al., 2021; Zhang et al., 2024).

Among the 12 key components identified by OPLS-DA, 6 of them showed relatively large contributions to sensory quality. Furan, pyran and lactone had a positive correlation with several sensory indicators other than aroma quality, stability and total score. This may partly explain why flue-cured tobacco and sun-cured yellow tobacco had better scores in residual and irritancy than sun-cured red tobacco. In contrast, 2-cyclopenten-1-one, 2,3-dimethyl- and 3-ethyl-2-hydroxy-2-cyclopenten-1-one had negative associations with several negative sensory indicators, which agrees with previous studies (Lu et al., 2025). Among organic acids, 3-methylvaleric acid showed negative effects on dry sensation (r = -0.241), bitterness (r = -0.183) and residual (r = -0.381), suggesting that appropriate levels of organic acids in the aerosol can alleviate negative mouthfeel and aftertaste.

Overall, both desirable and undesirable sensory characteristics of HTPs are governed by a limited subset of smoke components, particularly certain furan derivatives, phenols, ketones and organic acids.

### 4.3 The differences in sensory characteristics among different types of tobacco leaves

Flue-cured tobacco had the highest scores for residual (7.04 ± 0.44), bitterness (6.79 ± 0.36), dry sensation (6.78 ± 0.40), irritancy (6.87 ± 0.48) and strength (6.47 ± 0.77), indicating a relatively clean and soft taste and a more satisfying smoking experience. Sun-cured yellow tobacco showed more aroma concentration (6.60 ± 0.52) and offensive odor (6.52 ± 0.32), and its taste was rougher compared to flue-cured tobacco. Sun-cured red tobacco had the lowest aroma quality (6.22 ± 0.33), residual (6.07 ± 0.47), bitterness (6.07 ± 0.55), dry sensation (6.20 ± 0.39), irritancy (6.16 ± 0.63) and offensive odor (6.16 ± 0.30) score. Overall, flue-cured tobacco had the best palatability, sun-cured yellow tobacco had the richest aroma concentration, and sun-cured red tobacco had the strongest irritancy.

## 5 Conclusion

In this study, OPLS-DA, CCA and related chemometric methods were used to investigate the relationships between tobacco leaf powder aroma constituents, heated tobacco smoke components and sensory quality for flue-cured tobacco, sun-cured yellow tobacco and sun-cured red tobacco. A number of key components that significantly influence HTPs sensory quality and their directions of effect were identified.

CCA showed that 2-cyclopenten-1-one, 2,3-dimethyl-, 3-ethyl-2-hydroxy-2-cyclopenten-1-one, palmitic acid and chlorogenic acid made positive correlated with the sensory quality of HTPs, whereas dihydroactinidiolide, 2-furaldehyde, furfuryl alcohol and several other components had a positively correlated with negative sensory indicators such as dry sensation, bitterness and residual. Flue-cured tobacco contained higher levels of palmitic acid, chlorogenic acid and 2-cyclopenten-1-one, 2,3-dimethyl-, had the best overall sensory quality and the cleanest and softest smoking experience. Sun-cured yellow tobacco contained more palmitic acid and 3-ethyl-2-hydroxy-2-cyclopenten-1-one and provided the richest aroma, but the taste is relatively rough. Sun-cured red tobacco had higher dihydroactinidiolide and more nitrogen-containing components and yielded the poorest sensory experience, with lower aroma quality and stronger irritancy, dry sensation, bitterness and residual.

When conducting the correlation analysis, we found that there may be interactions among key components, which led to slight differences between the results of Pearson correlation analysis and canonical correlation analysis. However, canonical correlation analysis is more systematic, holistic and macroscopic, so the results of canonical correlation analysis were adopted in the subsequent analysis. But this also provides a new idea for exploring the key aroma components of heated tobacco products. Aroma components do not merely provide positive or negative influences singly, but whether there is an optimal content threshold. In addition, in the CCA of tobacco leaf powder aroma components and heated tobacco smoke components, only the first pair of canonical correlation variables reached the significance level (p<0.05), but the correlation coefficients of the second pair of canonical correlation variables (r=0.791; r=0.768) still had a certain magnitude in numerical terms, indicating that there might be a relatively weak secondary correlation. However, due to the limitation of the sample size in this study, further exploration could not be conducted. Nevertheless, this provides a potential exploration direction for future studies using a large sample size.

This study clarifies overall correlations between aroma components and sensory quality of HTPs but does not resolve threshold effects or transformation mechanisms of aroma components. Future research can focus on targeted experiments to determine optimal concentration ranges and interaction effects among key markers.

## Data Availability

The original contributions presented in the study are included in the article/**Supplementary Material**. Further inquiries can be directed to the corresponding author.
