# Supplementary material for: Key aroma and smoke components influencing sensory quality of heated tobacco products
Source: Front Plant Sci. 2026 May 4;17:1824692. doi: 10.3389/fpls.2026.1824692 (PMC13180813; doi:10.3389/fpls.2026.1824692)
Supplement: Supplementary file 1 [file Table1.docx]

**Supplementary Information**

Table A1 Detailed information of tobacco leaf powder the aroma components

| NO. | Components | CAS | Molecular Formula | Relative Molecular Mass |
| --- | --- | --- | --- | --- |
| Organic Acids | | | | |
| 1 | Malic Acid | 97-67-6 | C_4_H_6_O_5_ | 134.09 |
| 2 | Oxalic Acid | 144-62-7 | C_2_H_2_O_4_ | 90.03 |
| 3 | Citric Acid | 77-92-9 | C_6_H_8_O_7_ | 192.12 |
| 4 | Propanedioic Acid | 141-82-2 | C_3_H_4_O_4_ | 104.06 |
| 5 | Lactic Acid | 50-21-5 | C_3_H_6_O_3_ | 90.08 |
| 6 | Palmitic Acid | 57-10-3 | C_16_H_32_O_2_ | 256.42 |
| 7 | Stearic Acid | 57-11-4 | C_18_H_36_O_2_ | 284.48 |
| 8 | Oleic Acid | 112-80-1 | C_18_H_34_O_2_ | 282.46 |
| 9 | Linoleic Acid | 60-33-3 | C_18_H_32_O_2_ | 280.44 |
| Polyphenol | | | | |
| 10 | Chlorogenic Acid | 327-97-9 | C_16_H_18_O_9_ | 354.31 |
| 11 | Scopoletin | 92-61-5 | C_10_H_8_O_4_ | 192.17 |
| 12 | Rutin | 153-18-4 | C_27_H_30_O_16_ | 610.52 |
| Pigment | | | | |
| 13 | Lutein | 127-40-2 | C_40_H_56_O_2_ | 568.87 |
| 14 | β-Carotene | 7235-40-7 | C_40_H_56_ | 536.88 |
| Neutral Aroma Components | | | | |
| 15 | Solanone | 1937-54-8 | C_13_H_22_O | 194.31 |
| 16 | Geranyl Acetone | 3796-70-1 | C_13_H_22_O | 194.32 |
| 17 | β-Ionone | 14901-07-6 | C_13_H_20_O | 192.30 |
| 18 | 4-Keto-α-Ionone | [27185-77-9](https://www.chemsrc.com/baike/1329433.html) | C_13_H_18_O_2_ | 206.28 |
| 19 | Norsolanadione | 60619-46-7 | C_12_H_20_O_2_ | 196.29 |
| 20 | 3-Hydroxy-β-Damascone | 35734-61-3 | C_13_H_20_O_2_ | 208.30 |
| 21 | 3-oxo-α-Ionone | 49816-95-7 | C_13_H_18_O_2_ | 206.29 |
| 22 | Megastigmatrienone | 13215-88-8 | C_13_H_18_O | 190.28 |
| 23 | Dihydroactinidiolide | 15356-74-8 | C_11_H_16_O_2_ | 180.24 |
| 24 | Neophytadiene | 504-96-1 | C_20_H_38_ | 278.52 |

Table A2 Detailed information of tobacco leaf powder the aroma components

| NO. | Components | CAS | Molecular Formula | Relative Molecular Mass |
| --- | --- | --- | --- | --- |
| Furan, Pyran and Lactone | | | | |
| 1 | 2-Furaldehyde | 98-01-1 | C_5_H_4_O_2_ | 96.08 |
| 2 | Furfuryl Alcohol | 98-00-0 | C_5_H_6_O_2_ | 98.10 |
| 3 | 5-Methyl Furfural | 620-02-0 | C_6_H_6_O_2_ | 110.11 |
| 4 | 4-Hydroxy-2,5-Dimethyl-3(2H)-Furanone | 3658-77-3 | C₆H₈O₃ | 128.13 |
| 5 | Maltol | 118-71-8 | C_6_H_6_O_3_ | 126.11 |
| 6 | DDMP | 28564-83-2 | C_6_H_8_O_4_ | 144.13 |
| 7 | γ-Crotonolactone | 497-23-4 | C_4_H_4_O_2_ | 84.07 |
| 8 | Dihydroactinidiolide | 15356-74-8 | C_11_H_16_O_2_ | 180.24 |
| 9 | γ-Butyrolactone | 96-48-0 | C_4_H_6_O_2_ | 86.09 |
| Phenols | | | | |
| 10 | Phenol | 108-95-2 | C_6_H_5_OH | 94.11 |
| 11 | Guaiacol | 90-05-1 | C_7_H_8_O_2_ | 124.13 |
| 12 | o-Cresol | 95-48-7 | C_7_H_8_O | 108.14 |
| 13 | m-Cresol | 108-39-4 | C_7_H_8_O | 108.14 |
| 14 | p-Cresol | 106-44-5 | C_7_H_8_O | 108.14 |
| 15 | 4-Vinylguaiacol | 7786-61-0 | C_9_H_10_O_2_ | 150.17 |
| 16 | 2,6-Dimethylphenol | 576-26-1 | C_8_H_10_O | 122.16 |
| 17 | 2,6-Dimethoxyphenol | 91-10-1 | C_8_H_10_O_3_ | 154.17 |
| 18 | Eugenol | 97-53-0 | C_10_H_12_O_2_ | 164.20 |
| 19 | Isoeugenol | 97-54-1 | C_10_H_12_O_2_ | 164.20 |
| Ketones and Acids | | | | |
| 20 | 2-Cyclopentenone | 930-30-3 | C_5_H_6_O | 82.10 |
| 21 | 3-Methyl-2-Cyclopenten-1-One | 2758-18-1 | C_6_H_8_O | 96.13 |
| 22 | 2-Cyclopenten-1-One,2,3-Dimethyl- | 1121-05-7 | C_7_H_10_O | 110.15 |
| 23 | 3-Ethyl-2-Cyclopenten-1-One | [5682-69-9](https://www.lookchem.cn/cas_5682-69-9.html) | C_7_H_10_O | 110.16 |
| 24 | Methylcyclopentenolone | 80-71-7 | C_6_H_8_O_2_ | 112.13 |
| 25 | 3-Ethyl-2-Hydroxy-2-Cyclopenten-1-One | 21835-01-8 | C_7_H_10_O_2_ | 126.15 |
| 26 | 1-Indanone | 83-33-0 | C_9_H_8_O | 132.16 |
| 27 | Megastigmatrienone | 13215-88-8 | C_13_H_18_O | 190.28 |
| 28 | 3-Hydroxy-β-Damascone | 35734-61-3 | C_13_H_20_O_2_ | 208.30 |
| 29 | 3-oxo-α-Ionone | 49816-95-7 | C_13_H_18_O_2_ | 206.29 |
| 30 | Acetic Acid | 64-19-7 | CH_3_COOH | 60.05 |
| 31 | Propionic acid | 79-09-4 | CH_3_CH_2_COOH | 74.08 |
| 32 | Butyric Acid | 107-92-6 | CH_3_(CH_2_)_2_COOH | 88.11 |
| 33 | Isovaleric Acid | 503-74-2 | C_5_H_10_O_2_ | 102.12 |
| 34 | 3-Methylvaleric Acid | 105-43-1 | C_6_H_12_O_2_ | 116.16 |
| Nitrogen-containing Compounds | | | | |
| 35 | Pyrrole | 109-97-7 | C_4_H_5_N | 67.09 |
| 36 | 2-Acetylpyrrole | 1072-83-9 | C_6_H_7_NO | 109.13 |
| 37 | 2-Pyrrolidinone | 616-45-5 | C_4_H_7_NO | 85.10 |

Continue Table A2 Detailed information of tobacco leaf powder the aroma components

| 38 | pyridine | 110-86-1 | C_5_H_5_N | 79.10 |
| --- | --- | --- | --- | --- |
| 39 | 2-Methylpyridine | 109-06-8 | C_6_H_7_N | 93.13 |
| 40 | 3-Methylpyridine | 108-99-6 | C_6_H_7_N | 93.13 |
| 41 | 3-Ethylpyridine | 536-78-7 | C_7_H_9_N | 107.15 |
| 42 | 3-Hydroxy-6-Methylpyridine | 1121-78-4 | C_6_H_7_NO | 109.13 |
| 43 | 2,3'-Bipyridine | 581-50-0 | C_10_H_8_N_2_ | 156.18 |
| 44 | Nicotine | 54-11-5 | C_10_H_14_N_2_ | 162.23 |
| 45 | Myosmine | 532-12-7 | C_9_H_10_N_2_ | 146.19 |
| 46 | Nicotyrine | 487-19-4 | C_10_H_10_N_2_ | 158.21 |
| 47 | 2-Methylpyrazine | 109-08-0 | C_5_H_6_N_2_ | 94.12 |
| 48 | Indole | 120-72-9 | C_8_H_7_N | 117.15 |
| 49 | 3-Methylindole | 83-34-1 | C_9_H_9_N | 131.18 |
| 50 | Quinoline | 91-22-5 | C_9_H_7_N | 129.16 |
| 51 | Isovaleramide | 541-46-8 | C_5_H_11_NO | 101.15 |
| Other Components | | | | |
| 52 | Neophytadiene | 504-96-1 | C_20_H_38_ | 278.52 |
| 53 | Benzyl Alcohol | 100-51-6 | C_7_H_8_O | 108.14 |
| 54 | Phenethyl Alcohol | 60-12-8 | C_8_H_10_O | 122.17 |
